# Suppressing Effect of Free Triiodothyronine on the Negative Association between Body Mass Index and Serum Osteocalcin Levels in Euthyroid Population

**DOI:** 10.1155/2021/6624516

**Published:** 2021-02-05

**Authors:** Xiaomin Nie, Yiting Xu, Yun Shen, Yufei Wang, Xiaojing Ma, Yuqian Bao

**Affiliations:** Department of Endocrinology and Metabolism, Shanghai Jiao Tong University Affiliated Sixth People's Hospital, Shanghai Clinical Center for Diabetes, Shanghai Key Clinical Center for Metabolic Disease, Shanghai Diabetes Institute, Shanghai Key Laboratory of Diabetes Mellitus, Shanghai 200233, China

## Abstract

Previous studies found that thyroid hormones stimulate osteoblast-like cells to secrete osteocalcin. We aimed to investigate the association between serum thyroid hormone and serum osteocalcin in euthyroid population. The study recruited 1152 community-based euthyroid subjects (average age 59 ± 8 years), among whom 677 were women. Serum free triiodothyronine (FT3), free thyroxine (FT4), thyroid-stimulating hormone (TSH), and osteocalcin were measured by electrochemiluminescence immunoassays. After adjusting for age and gender, partial correlation analysis showed that FT3 and FT3/FT4 were both positively correlated with body mass index (BMI) and serum osteocalcin levels (all *P* < 0.05) and BMI was negatively correlated with serum osteocalcin levels (*P* < 0.01), while FT4 and TSH were not correlated with serum osteocalcin levels (all *P* > 0.05). Age, gender, blood pressure, thyroid hormones, and multiple metabolic risk factors were included in the ridge regression model. FT3 and FT3/FT4 were independently and positively associated with serum osteocalcin levels (all *P* < 0.05), while BMI was independently and negatively associated with serum osteocalcin levels (*P* < 0.01). The mediating effect model showed that FT3 and FT3/FT4 suppressed the negative association between BMI and serum osteocalcin levels, with suppressing effects of 6.41% and 10.39%, respectively. In euthyroid subjects, both FT3 and FT3/FT4 were positively associated with serum osteocalcin levels, and they further suppressed the negative association between BMI and serum osteocalcin levels.

## 1. Introduction

Endocrine functions of the bone have attracted extensive attention in recent years. The bone influences energy metabolism via secreting various cytokines and hormones, among which osteocalcin, specifically secreted by osteoblasts and osteocytes, plays an important role [1]. Osteocalcin is encoded by the human gene *BGLAP* and is the most abundant noncollagen protein in the bone marrow [2]. As a marker of bone remodeling, osteocalcin regulates the size and shape of bone mineral substances in the vitamin K-dependent *γ*-carboxylated form [3]. Outside the bone, osteocalcin promotes islet *β* cell proliferation, stimulates insulin secretion, and regulates adipokine expression to influence energy metabolism [4]. The production and secretion of osteocalcin are regulated by local and humoral factors [2].

Thyroid hormones are important for bone development, maturation, and metabolism [5]. Different types of thyroid hormone receptors are expressed in human osteoblasts [6]. Previous studies found that thyroid hormones regulated the proliferation and differentiation of osteoblasts and the expression of bone turnover factors including osteocalcin [7]. In clinical studies, serum osteocalcin levels were found to increase in hyperthyroidism [8, 9], decrease in hypothyroidism [10], and recover to normal levels after treatment [11, 12]. Thyroid hormones and osteocalcin both play important roles in energy metabolism; however, whether circulating thyroid hormones are associated with serum osteocalcin levels remains unclear. Previous studies in Australian and German populations did not reach consensus on the association between serum thyroid hormone levels and serum osteocalcin levels [13, 14], in which the bioactive form of thyroid hormone, free triiodothyronine (FT3), was not tested. At present, no study has explored the association between serum thyroid hormones and osteocalcin levels in a Chinese population. In this study, we aimed to explore the associations between serum thyroid hormone levels and serum osteocalcin levels in a community-based euthyroid population and to investigate the potential role of serum thyroid hormone levels in the association between body mass index (BMI) and serum osteocalcin levels.

## 2. Materials and Methods

### 2.1. Subjects

This study recruited 1152 community-based subjects from October 2015 to July 2016 in Shanghai [15]. Postmenopausal status was defined as at least 1 year of amenorrhea in the absence of other medical conditions [16]. All subjects completed a questionnaire survey and underwent a physical examination and laboratory measurements. The study included Han Chinese adults who volunteered to participate in the study and were able to provide the information required for the survey. According to the 2001 International Physical Activity Questionnaire (IPAQ), the levels of physical activity are classified as light, moderate, or heavy [17]. The exclusion criteria included a valid history of diabetes or cardiovascular diseases, moderate to severe anemia, malignancy, severe hepatic or renal dysfunction, acute infection, antithyroid therapies, abnormal thyroid function, hormone replacement treatment such as thyroid hormones or sex hormones, bone fracture, and use of hypotensive drugs, lipid-regulating drugs, glucocorticoids, lithium, amiodarone, or other medications that affect bone metabolism. All participants signed informed consent forms. The study was approved by the Ethics Committee of the Shanghai Jiao Tong University Affiliated Sixth People's Hospital and was conducted in accordance with the Declaration of Helsinki.

### 2.2. Body Measurements and Laboratory Examinations

Height and body weight were measured according to standard methods described in our previous study [15], BMI = body weight (kg)/height^2^ (m^2^). Systolic blood pressure (SBP) and diastolic blood pressure (DBP) were measured as the mean of 3 blood pressure measurements taken at 3-minute intervals.

All subjects underwent a 75-g oral glucose tolerance test in the morning after an overnight fast of 10 hours. Fasting plasma glucose (FPG), 2-hour plasma glucose (2hPG), total cholesterol (TC), triglyceride (TG), high-density lipoprotein cholesterol (HDL-c), low-density lipoprotein cholesterol (LDL-c), and glycated hemoglobin A1c (HbA1c) were detected by methods described previously [15]. Serum FT3, free thyroxine (FT4), and thyroid-stimulating hormone (TSH) levels were measured by electrochemiluminescence immunoassays (Roche Diagnostics GmbH, Mannheim, Germany) on a Cobas e601 analyzer. Serum osteocalcin levels were measured by electrochemiluminescence immunoassays (Roche Diagnostics GmbH, Mannheim, Germany) with intra-assay coefficients of variation 1.2–4.0% and interassay coefficients of variation 1.7–6.5%.

### 2.3. Statistical Analyses

All data analyses were performed by using SPSS version 22.0 (SPSS, Inc., Chicago, IL, USA) statistical software. A two-tailed *P* value <0.05 was considered statistically significant. The Kolmogorov–Smirnov test was used to evaluate the normal distribution of all continuous variables. Variables with a normal distribution are expressed as the means ± standard deviations. Skewed distributed variables were expressed as medians (interquartile ranges). Categorical variables were expressed as numbers (percentages). Partial correlation analysis was used to explore the correlations among serum thyroid hormone levels, serum osteocalcin levels, and BMI after adjusting for age and gender. Ridge regression analysis was used to explore the associations between serum thyroid hormone levels and serum osteocalcin levels after adjusting for confounding factors. Based on a mediating effect model, the suppressing effect was evaluated to investigate the influence of serum thyroid hormone levels on the associations between BMI and serum osteocalcin levels [18].

## 3. Results

### 3.1. Clinical Characteristics of Study Subjects

The study included 1152 subjects (age range: 27–81 years; average 59 ± 8 years). There were 475 men and 677 women. Among the women, 96 were eumenorrheic and 581 were postmenopausal. The clinical characteristics of the study subjects are shown in Table 1. The median (interquartile range) serum osteocalcin level was 19.44 (15.69–24.78) ng/mL, and women had higher serum osteocalcin levels than men (20.91 (16.88–25.43) vs. 17.27 (14.55–21.30) ng/mL, *P* < 0.01).

### 3.2. Partial Correlation Analysis among Serum Thyroid Hormone Levels, Serum Osteocalcin Levels, and BMI

After adjusting for age and gender (Table 2), FT3 was positively correlated with FT4 (*r* = 0.190), FT3/FT4 (*r* = 0.537), serum osteocalcin (*r* = 0.104), and BMI (*r* = 0.080) (all *P* < 0.05), but it was not correlated with TSH (*P* > 0.05). FT4 was negatively correlated with FT3/FT4 (*r* = −0.716), TSH (*r* = −0.182), and BMI (*r* = −0.111) (all *P* < 0.05), but it was not correlated with serum osteocalcin levels (*P* > 0.05). FT3/FT4 was positively correlated with TSH (*r* = 0.164), serum osteocalcin levels (*r* = 0.091), and BMI (*r* = 0.157) (all *P* < 0.05). TSH was not correlated with serum osteocalcin levels or BMI (all *P* > 0.05). Serum osteocalcin levels were negatively correlated with BMI (*r* = −0.171, *P* < 0.01).

### 3.3. Ridge Regression Analysis of the Influencing Factors of Serum Osteocalcin Levels

**Figure 1:**
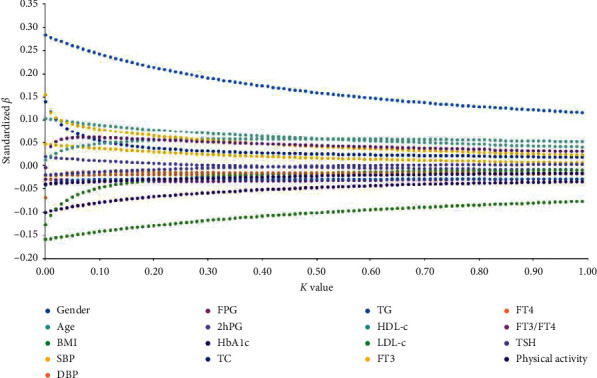
Ridge trace figure for the influencing factors of serum osteocalcin levels.

Ridge regression analysis was used to explore the influencing factors of serum osteocalcin levels. Independent variables included gender, age, BMI, SBP, DBP, FPG, 2hPG, HbA1c, TC, TG, HDL-c, LDL-c, FT3, FT4, FT3/FT4, TSH, and the levels of physical activity. The ridge trace figure (Figure 1) indicated that the standardized *β* of serum thyroid hormone levels tended to be steady when *K* = 0.4.

As shown in Table 3, female sex, age, HDL-c, FT3, and FT3/FT4 were all positively associated with serum osteocalcin levels (all *P* < 0.05). BMI and HbA1c were negatively associated with serum osteocalcin levels (all *P* < 0.05). However, SBP, DBP, FPG, 2hPG, TC, TG, LDL-c, FT4, TSH, and the levels of physical activity were not associated with serum osteocalcin levels (all *P* > 0.05).

### 3.4. Suppressing Effect of FT3 on the Association between Serum Osteocalcin Levels and BMI

**Figure 2:**
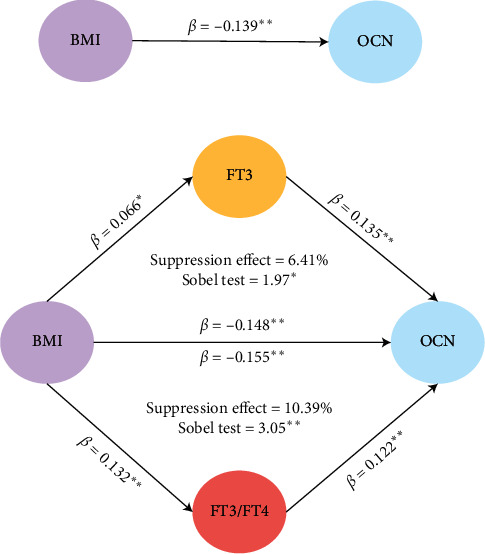
Suppressing effect of FT3 and FT3/FT4 on the association between BMI and serum osteocalcin levels. ^*∗∗*^*P* < 0.05, ^*∗∗*^*P* < 0.01. Age, gender, HbA1c, and HDL-c were adjusted for all models.

A mediating effect model was further used to investigate the influence of FT3 and FT3/FT4 on the association between serum osteocalcin levels and BMI (Figure 2). Age, gender, HbA1c, and HDL-c were adjusted. FT3 and FT3/FT4 suppressed the negative association between BMI and serum osteocalcin levels, with suppressing effects of 6.41% (Sobel test = 1.97, *P* < 0.05) and 10.39% (Sobel test = 3.05, *P* < 0.01), respectively.

## 4. Discussion

The present study explored the associations between serum thyroid hormone levels and serum osteocalcin levels in a community-based euthyroid population. We found that FT3 and FT3/FT4 were positively associated with serum osteocalcin levels, while FT4 and TSH were not associated with serum osteocalcin levels. FT3 and FT3/FT4 suppressed the negative association between BMI and serum osteocalcin levels.

Thyroid hormones contribute greatly to bone metabolism. Hyperthyroidism leads to accelerated bone turnover and decreased bone mass and is one of the clinical causes of secondary osteoporosis [19]. Conversely, hypothyroidism leads to dysplasia of bone maturation and epiphyseal development, thus further leading to growth retardation and skeletal deformity [20]. Serum osteocalcin levels in patients with hyperthyroidism were significantly higher than those of healthy controls, while they were significantly lower in patients with hypothyroidism. In patients with hypothyroidism, serum osteocalcin recovered to normal levels after thyroxine replacement treatment [12]. Siru et al. recruited 3338 elderly Australian men, among whom 3117 were euthyroid, 135 were subclinical hypothyroid, and 86 were subclinical hyperthyroid. They found that there were no differences in serum osteocalcin levels among the three groups, and there were no associations of FT4 or TSH with serum osteocalcin levels in euthyroid subjects [13]. In a German cohort of 2654 subjects, there was a nonlinear U-shaped curve relationship between TSH and serum osteocalcin levels in women, while no association was found between TSH and osteocalcin in men [14]. Similar to the results of Siru et al., we also found no associations of FT4 or TSH with serum osteocalcin levels in euthyroid subjects.

Previous studies found that triiodothyronine (T3) regulated the proliferation and differentiation of osteoblasts and the expression of various bone turnover factors including osteocalcin [7, 21]. FT3 is the bioactive form of thyroid hormones; however, whether FT3 influences serum osteocalcin in euthyroid subjects remains unknown. FT3 was not detected in the two previous studies in Australian and German populations. In this study, we investigated the associations of FT3 and FT3/FT4 with serum osteocalcin. A previous study found that osteocalcin levels increase with exercise interventions [22], and physical activity was included as a confounding factor in the regression analysis. Consistent with the results of previous cell experiments, FT3 and FT3/FT4 were significantly and positively associated with serum osteocalcin, suggesting that FT3 and FT3/FT4 may influence serum osteocalcin in euthyroid population. In addition, we used ridge regression analysis instead of traditional multivariate linear regression analysis to explore the independent influencing factors of serum osteocalcin levels. According to the results of partial correlation analysis, we found that FT3, FT4, and FT3/FT4 were significantly correlated with each other. The principle of a ridge regression analysis allowed us to simultaneously process variables with collinearity in one regression model, by which we could observe the independent influences of FT3, FT4, and FT3/FT4 on serum osteocalcin levels.

The potential association mechanism of serum thyroid hormone and serum osteocalcin has been explored in many previous studies. Thyroid hormone receptors were found to be expressed in both the rat osteogenic-like cell line ROS 17/2.8 and the mouse osteogenic-like cell line MC3T3-E1. T3 stimulated the secretion of osteocalcin in vitro [23, 24]. In vivo, the mRNA expression of osteocalcin in the femur of rats increased in a dose-dependent manner with T3 injection [25]. The thyroid hormone receptor TR*β*1 and sympathetic adrenal receptor *β*2 signaling pathways play a key role in mediating T3 to stimulate osteocalcin secretion [26, 27]. In the bone, DIO2 and DIO3 synergistically regulate the thyroid hormone signaling [28], which may partly explain our finding that FT3/FT4 is positively associated with osteocalcin. Further studies are needed to elucidate the mechanisms by which adipose tissue, thyroid, and bone interact with each other.

Previous studies found that subjects with obesity and nonalcoholic fatty liver disease had higher FT3 and FT3/FT4 levels [29–31]. Whether high FT3 and FT3/FT4, which are associated with metabolic disorders, are risk factors or compensatory mechanisms remains controversial. In this study, we found that FT3 suppressed the negative association between BMI and serum osteocalcin levels, suggesting that the high FT3 within the reference range may alleviate the decline in serum osteocalcin levels in obesity. Osteocalcin is a marker of bone remodeling. The obesity-related high FT3 level may induce bone remodeling by regulating osteoblast activity, by which the bone experiences structural changes to adapt to weight gain. In addition, osteocalcin exerts protective effects on energy metabolism; thus, the obesity-related high FT3 may promote the secretion of osteocalcin to compensate for glucose and lipid metabolism.

Our study has some limitations. Firstly, we could not determine causality between serum thyroid hormone levels, serum osteocalcin levels, and the obesity index based on a cross-sectional study. Secondly, the study subjects were Han Chinese adults, and thus our results may not be generalizable to other ethnic populations. Thirdly, as potential confounding factors, gonadal hormones are not included in our study, which need to be explored in future studies.

## 5. Conclusions

In conclusion, FT3 and FT3/FT4 were both positively associated with serum osteocalcin levels in euthyroid subjects, indicating that FT3 may influence the secretion of osteocalcin in euthyroid subjects. Furthermore, FT3 and FT3/FT4 suppressed the negative association between BMI and serum osteocalcin levels, suggesting that obesity-related high FT3 levels may alleviate the decline in serum osteocalcin levels.

## Figures and Tables

**Table 1:** Clinical characteristics of subjects.

Variables	*N* = 1152
Men/women	475/677
Age, years	59 (54–64)
BMI (kg/m^2^)	23.30 (21.40–25.30)
SBP (mmHg)	128 (117–140)
DBP (mmHg)	77 (70–84)
FPG (mmol/L)	5.68 (5.35–6.09)
2hPG (mmol/L)	7.05 (5.76–8.65)
HbA1c, % (mmol/mol)	5.7 (39) [5.4 (36)–5.9 (41)]
TC (mmol/L)	5.36 (4.77–6.00)
TG (mmol/L)	1.34 (0.95–1.96)
HDL-c (mmol/L)	1.40 (1.19–1.66)
LDL-c (mmol/L)	3.25 (2.74–3.82)
FT3 (pmol/L)	4.93 (4.59–5.30)
FT4 (pmol/L)	16.41 ± 1.81
FT3/FT4	0.30 (0.28–0.33)
TSH (mIU/L)	2.33 (1.64–3.31)
OCN (ng/mL)	19.44 (15.69–24.78)

Physical activity	
Light, N (%)	167 (14.50)
Moderate, N (%)	671 (58.20)
Heavy, N (%)	314 (27.30)

*Note.* Normally distributed variables were expressed as means ± SD, and skewed distributed variables were expressed as medians (interquartile ranges). Abbreviations: 2hPG, 2-hour plasma glucose; BMI, body mass index; DBP, diastolic blood pressure; FPG, fasting plasma glucose; FT3, free triiodothyronine; FT3/FT4, free triiodothyronine to free thyroxine ratio; FT4, free thyroxine; HbA1c, glycated hemoglobin A1c; HDL-c, high-density lipoprotein cholesterol; LDL-c, low-density lipoprotein cholesterol; OCN, osteocalcin; SBP, systolic blood pressure; TC, total cholesterol; TG, triglyceride; TSH, thyroid-stimulating hormone.

**Table 2:** Partial correlation analysis among serum thyroid hormones levels, serum osteocalcin levels, and BMI.

Variables	FT3	FT4	FT3/FT4	TSH	OCN
FT4	0.190^*∗∗*^	—	—	—	—
FT3/FT4	0.537^*∗∗*^	−0.716^*∗∗*^	—	—	—
TSH	−0.003	−0.182^*∗∗*^	0.164^*∗∗*^	—	—
OCN	0.104^*∗∗*^	−0.023	0.091^*∗*^	−0.016	—
BMI	0.080^*∗∗*^	−0.111^*∗∗*^	0.157^*∗∗*^	0.051	−0.171^*∗∗*^

*Note.* Age and gender were adjusted. ^*∗*^*P* < 0.05, ^*∗∗*^*P* < 0.01. Abbreviations: BMI, body mass index; FT3, free triiodothyronine; FT3/FT4, free triiodothyronine to free thyroxine ratio; FT4, free thyroxine; OCN, osteocalcin; TSH, thyroid-stimulating hormone.

**Table 3:** Ridge regression analysis on the influencing factors of serum osteocalcin levels.

Variables	Standardized *β*	*T*	*P* value
Gender^a^	0.173	8.546	<0.001
Age	0.063	3.152	0.002
BMI	−0.110	−5.436	<0.001
SBP	0.019	0.978	0.328
DBP	−0.019	−0.985	0.325
FPG	−0.034	−1.694	0.091
2hPG	−0.003	−0.129	0.897
HbA1c	−0.053	−2.627	0.009
TC	0.027	1.752	0.080
TG	−0.030	−1.489	0.137
HDL-c	0.057	2.930	0.003
LDL-c	−0.020	−1.220	0.223
FT3	0.046	2.777	0.006
FT4	−0.017	−1.069	0.285
FT3/FT4	0.046	3.410	0.001
TSH	−0.004	−0.185	0.853
Physical activity^b^	−0.025	−1.268	0.205

*Note. K* value = 0.4, *F* = 9.002, *P* < 0.001. ^a^Men were coded as “0,” and women were coded as “1.” ^b^Light was coded as “1,” moderate was coded as “2,” and heavy was coded as “3.”

## Data Availability

The data used to support the findings of this study are available from the corresponding authors upon request.

## Abbreviations

2hPG: 2-hour plasma glucose
DBP: Diastolic blood pressure
fat%: Fat percentage
FPG: Fasting plasma glucose
FT3: Free triiodothyronine
FT4: Free thyroxine
FT3/FT4: Free triiodothyronine to free thyroxine ratio
HbA1c: Glycated hemoglobin A1c
HDL-c: High-density lipoprotein cholesterol
LDL-c: Low-density lipoprotein cholesterol
SBP: Systolic blood pressure
TC: Total cholesterol
TG: Triglyceride
TSH: Thyroid-stimulating hormone.

## References

[1] DiGirolamo D. J., Clemens T. L., Kousteni S. (2012). The skeleton as an endocrine organ. *Nature Reviews Rheumatology*.

[2] Dirckx N., Moorer M. C., Clemens T. L., Riddle R. C. (2019). The role of osteoblasts in energy homeostasis. *Nature Reviews Endocrinology*.

[3] Ducy P. (2011). The role of osteocalcin in the endocrine cross-talk between bone remodelling and energy metabolism. *Diabetologia*.

[4] Lee N. K., Sowa H., Hinoi E. (2007). Endocrine regulation of energy metabolism by the skeleton. *Cell*.

[5] Bassett J. H. D., Williams G. R. (2016). Role of thyroid hormones in skeletal development and bone maintenance. *Endocrine Reviews*.

[6] Siddiqi A., Parsons M. P., Lewis J. L., Monson J. P., Williams G. R., Burrin J. M. (2002). TR expression and function in human bone marrow stromal and osteoblast-like cells. *The Journal of Clinical Endocrinology & Metabolism*.

[7] Kassem M., Mosekilde L., Eriksen E. F. (1993). Effects of triiodothyronine on DNA synthesis and differentiation markers of normal human osteoblast-like cells in vitro. *Biochemistry and Molecular Biology International*.

[8] Akalin A., Colak Ö., Alatas Ö., Efe B. (2002). Bone remodelling markers and serum cytokines in patients with hyperthyroidism. *Clinical Endocrinology*.

[9] Shinkov A. D., Borissova A.-M. I., Kovatcheva R. D., Atanassova I. B., Vlahov J. D., Dakovska L. N. (2014). Age and menopausal status affect osteoprotegerin and osteocalcin levels in women differently, irrespective of thyroid function. *Clinical Medicine Insights Endocrinology and Diabetes*.

[10] Botella-Carretero J. I., Alvarez-Blasco F., Millán J. L. S., Escobar-Morreale H. F. (2007). Thyroid hormone deficiency and postmenopausal status independently increase serum osteoprotegerin concentrations in women. *European Journal of Endocrinology*.

[11] Garnero P., Vassy V., Bertholin A., Riou J. P., Delmas P. D. (1994). Markers of bone turnover in hyperthyroidism and the effects of treatment. *The Journal of Clinical Endocrinology & Metabolism*.

[12] Kojima N., Sakata S., Nakamura S. (1992). Serum concentrations of osteocalcin in patients with hyperthyroidism, hypothyroidism and subacute thyroiditis. *Journal of Endocrinological Investigation*.

[13] Siru R., Alfonso H., Chubb S. A. P., Golledge J., Flicker L., Yeap B. B. (2018). Subclinical thyroid dysfunction and circulating thyroid hormones are not associated with bone turnover markers or incident hip fracture in older men. *Clinical Endocrinology*.

[14] Tsourdi E., Wallaschofski H., Rauner M. (2016). Thyrotropin serum levels are differentially associated with biochemical markers of bone turnover and stiffness in women and men: results from the SHIP cohorts. *Osteoporosis International*.

[15] Xu Y., Ma X., Pan X., He X., Xiao Y., Bao Y. (2018). Correlations between serum concentration of three bone-derived factors and obesity and visceral fat accumulation in a cohort of middle aged men and women. *Cardiovascular Diabetology*.

[16] National Collaborating Centre for Ws (2015). *Children’s H. National Institute for Health and Care Excellence: Clinical Guidelines. Menopause: Full Guideline*.

[17] Craig C. L., Marshall A. L., Sjöström M. (2003). International physical activity questionnaire: 12-country reliability and validity. *Medicine & Science in Sports & Exercise*.

[18] Kenny D. A., Korchmaros J. D., Bolger N. (2003). Lower level mediation in multilevel models. *Psychological Methods*.

[19] Stern P. H., Bilezikian J. P., Raisz L. G., Rodan G. A. (2002). Chapter 40-thyroid hormone and bone. *Principles of Bone Biology*.

[20] Allain T. J., McGregor A. M. (1993). Thyroid hormones and bone. *Journal of Endocrinology*.

[21] Langdahl B. L., Loft A. G., Møller N. (1997). Is skeletal responsiveness to thyroid hormone altered in primary osteoporosis or following estrogen replacement therapy?. *Journal of Bone and Mineral Research*.

[22] Mohammad Rahimi G. R., Niyazi A., Alaee S. (2020). The effect of exercise training on osteocalcin, adipocytokines, and insulin resistance: a systematic review and meta-analysis of randomized controlled trials. *Osteoporosis International*.

[23] Gouveia C., Schultz J., Bianco A., Brent G. (2001). Thyroid hormone stimulation of osteocalcin gene expression in ROS 17/2.8 cells is mediated by transcriptional and post-transcriptional mechanisms. *Journal of Endocrinology*.

[24] Kasono K., Sato K., Han D. C., Fujii Y., Tsushima T., Shizume K. (1988). Stimulation of alkaline phosphatase activity by thyroid hormone in mouse osteoblast-like cells (MC3T3-E1): a possible mechanism of hyperalkaline phosphatasia in hyperthyroidism. *Bone and Mineral*.

[25] Ross D. S., Graichen R. (1991). Increased rat femur osteocalcin mRNA concentrations following in vivo administration of thyroid hormone. *Journal of Endocrinological Investigation*.

[26] Beber E. H., Capelo L. P., Fonseca T. L. (2009). The thyroid hormone receptor (TR) *β*-selective agonist GC-1 inhibits proliferation but induces differentiation and TR *β* mRNA expression in mouse and rat osteoblast-like cells. *Calcified Tissue International*.

[27] Neofiti-Papi B., Albuquerque R. P., Miranda-Rodrigues M. (2019). Thyrotoxicosis involves *β*2-adrenoceptor signaling to negatively affect microarchitecture and biomechanical properties of the femur. *Thyroid*.

[28] Capelo L. P., Beber E. H., Huang S. A., Zorn T. M. T., Bianco A. C., Gouveia C. H. A. (2008). Deiodinase-mediated thyroid hormone inactivation minimizes thyroid hormone signaling in the early development of fetal skeleton. *Bone*.

[29] Taylor P. N., Richmond R., Davies N. (2016). Paradoxical relationship between body mass index and thyroid hormone levels: a study using mendelian randomization. *The Journal of Clinical Endocrinology & Metabolism*.

[30] van den Berg E. H., van Tienhoven-Wind L. J. N., Amini M. (2017). Higher free triiodothyronine is associated with non-alcoholic fatty liver disease in euthyroid subjects: the Lifelines Cohort Study. *Metabolism*.

[31] Michalaki M. A., Vagenakis A. G., Leonardou A. S. (2006). Thyroid function in humans with morbid obesity. *Thyroid*.

